# Aetiology of neonatal sepsis in Nigeria, and relevance of *Group b streptococcus*: A systematic review

**DOI:** 10.1371/journal.pone.0200350

**Published:** 2018-07-17

**Authors:** Nubwa Medugu, Kenneth Iregbu, Pui-Ying Iroh Tam, Stephen Obaro

**Affiliations:** 1 Department of Medical Microbiology and Parasitology, National Hospital Abuja, Abuja, Nigeria; 2 Malawi-Liverpool Wellcome Trust Clinical Research Programme, Blantyre, Malawi; 3 Division of Paediatric Infectious Diseases, University of Nebraska Medical Center, Omaha, Nebraska, United States of America; Public Health England, UNITED KINGDOM

## Abstract

**Background:**

Group B Streptococcus (GBS) causes invasive infections in neonates and has been implicated as a cause of prelabour rupture of membranes, preterm delivery and stillbirths. The success of phase II trials of polyvalent polysaccharide GBS vaccines indicates that these infections are potentially preventable. Nigeria is the most populous country in Africa with one of the highest birth rates, one of the highest neonatal sepsis incidence rates and one of the highest mortality rates in the world. Therefore, before the possible introduction of preventive strategies such as intrapartum antibiotic prophylaxis or GBS vaccine into Nigeria, it is vital that there is accurate data on the aetiology of neonatal sepsis and on the incidence of GBS neonatal sepsis in particular. The objective of this study was to determine the incidence and aetiology of neonatal sepsis in Nigeria with a focus on GBS sepsis and also to assess the potential impact of a GBS vaccine.

**Methods:**

A literature search was conducted on the databases of African journals online, PubMed and Google Scholar for works conducted between 1987 to 2017. Case reports, reviews, and studies not stating specific culture methods or specific bacteria isolated were excluded. Data extracted included; incidence of neonatal sepsis, method of blood culture, blood volume, sample size, bacterial agents isolated and history of antibiotic use. PRISMA guidelines were followed and modified Down’s and Black criteria used to evaluate the quality of studies.

**Results:**

A total of 5,114 studies were reviewed for neonatal sepsis out of which 24 consisting of a total of 2,280 cases were selected for final review. Nine studies met criteria for assessment of hospital based incidence of neonatal sepsis representing 31,305 hospital births. The incidence of neonatal sepsis was 18.2/1000 livebirths with range from 7-55/1000 livebirths while the GBS incidence was 0.06/1000 livebirths with range from 0-2/1000 live births. We discovered various limitations such as identification techniques that could result in underestimation of the true incidence of GBS sepsis. Pathogens such as *Klebsiella pneumoniae* and *Staphylococcus aureus* were more commonly isolated than GBS.

**Implications of key findings:**

The hospital based incidence of neonatal sepsis was high at 18.2/1000 live births while that due to GBS was 0.06/1000 live births. The burden of neonatal sepsis, including that attributable to GBS is substantial and could be reduced by preventive strategies such as intrapartum antibiotic prophylaxis or GBS vaccine. There is however very sparse meaningful data currently. Well planned prospective studies with larger sample sizes, more advanced isolation and identification techniques and those following up invasive disease cases for possible short and long term sequelae are needed—not only prior to possible introduction of the vaccine to determine the baseline epidemiology, but also thereafter to monitor its impact on the population. Strategies need to be developed to also reduce the morbidity and mortality attributable to other bacteria that have an incidence even greater than that of GBS.

## Introduction

Group B Streptococcus (GBS) is a major cause of morbidity and mortality, and is one of the commonest causes of invasive infection among newborns worldwide[1–3]. While the incidence of newborn GBS sepsis in developing countries is estimated at 0-3/1000 live births[4], which is similar to the 1-2/1000 live births reported in the USA before initiation of intrapartum antibiotic prophylaxis (IAP) as a prevention measure[5], the exceedingly higher occurrence of neonatal sepsis from *S*. *aureus* and Enterobacteriaceae in these regions seem to overshadow this threat, with estimated incidence from 3–29/ 1000 live births[6,7]. Hence, in most of Africa and the developing world, GBS is not listed in the top three etiological agents of neonatal sepsis[8,9]. African countries like Kenya, South Africa, Zimbabwe and Malawi have however reported high rates of GBS sepsis[10–17]. Incidence rate in Malawi and South Africa is reported at 1.8 and 3/1000 live births respectively[11,18]. GBS was also reported as the commonest cause of neonatal meningitis in Malawi[19]. The reason for disparity of high incidence of GBS neonatal disease in certain African countries but not in others—like Nigeria—is not clear. The consequences of invasive GBS infections are diverse and severe for the newborn, family and economy. Nigeria is the most populous country in Africa and has one of the highest birth, neonatal sepsis and neonatal mortality rates in the world[20–22]. Data from this country is vital to inform healthcare policy and develop public health programs that target this vulnerable population. A preventive strategy which includes a GBS vaccine could therefore have an positive impact on the pattern of morbidity and mortality in the Nigerian population. It is therefore important that the burden of neonatal GBS sepsis in Nigeria be measured.

The goal of this study was to systematically review and summarize the studies so far conducted and answer questions about the incidence and etiological agents of neonatal sepsis in Nigeria and how much of that is attributable to GBS?

## Methods

### Data sources and search strategy

Studies were searched and identified using the stepwise approach specified in the Preferred Reporting Items for Systematic Reviews and Meta-Analyses (PRISMA) Statement [23]. All original studies addressing neonatal sepsis in Nigeria and published in English language between 1 January 1987 and 5 April 2017 were actively looked for. We searched on databases of African journals online (AJOL), PubMed and Google Scholar. We looked for manuscripts incorporating at least one of the following medical subject headings (MESH) in the title or abstract; Nigeria, sepsis, septicaemia, bacteraemia, neonatal, neonate, neonates. This is detailed in Table 1. Bibliographies were searched for potential studies using the snowball method.

**Fig 1:**
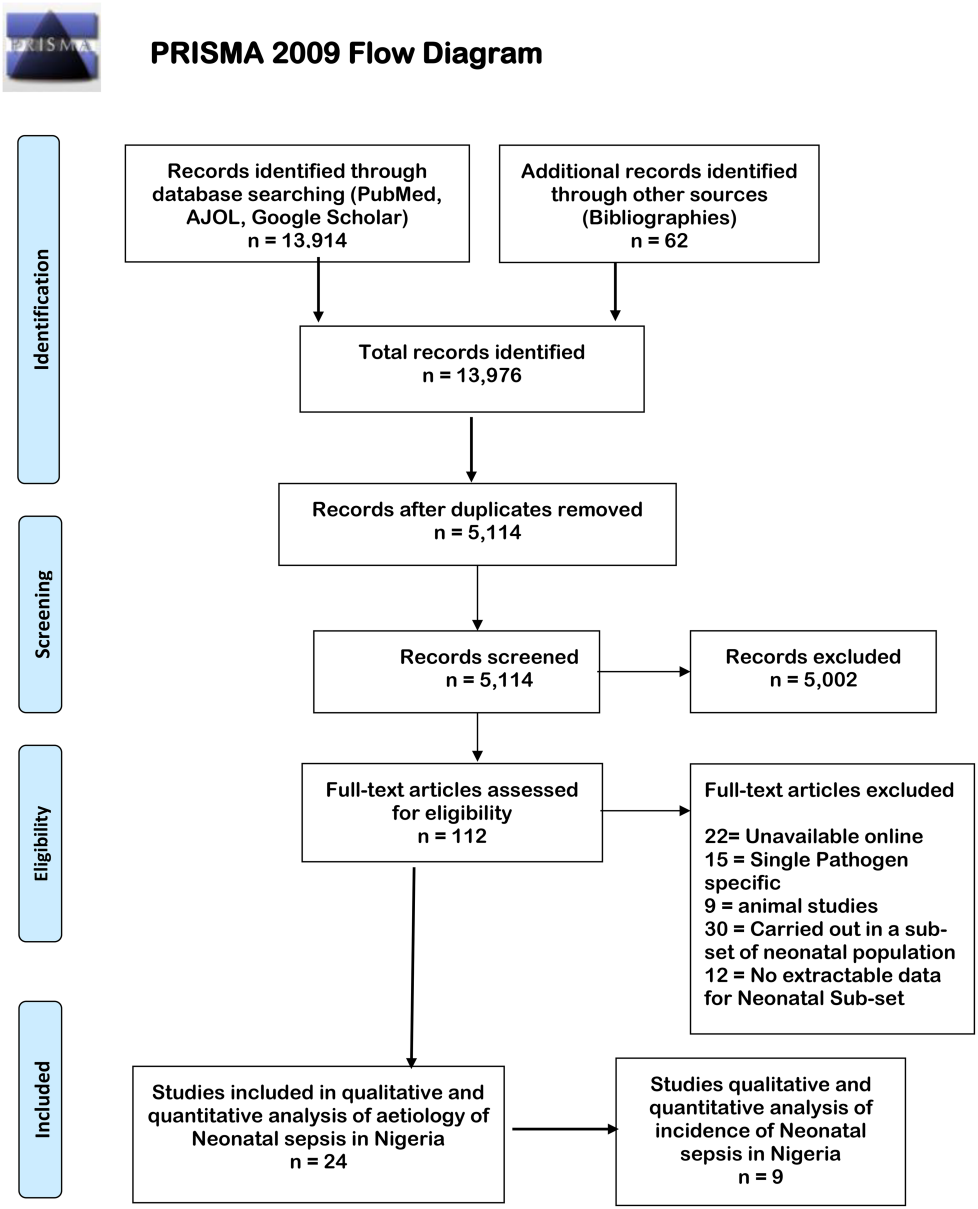
Flowchart depicting stages of article selection for evaluation of neonatal sepsis in Nigeria. *From*: Moher D, Liberati A, Tetzlaff J, Altman DG, The PRISMA Group (2009). *P*referred *R*eporting *I*tems for *S*ystematic Reviews and *M*eta-*A*nalyses: The PRISMA Statement. PLoS Med 6(7): e1000097. doi: 10.1371/journal.pmed1000097 **For more information, visit** www.prisma-statement.org.

We excluded case reports, case series, reviews, studies available only in abstract form, and studies specifying only a single agent of sepsis, e.g. ‘incidence of *Klebsiella pneumoniae* sepsis in a hospital’. Studies identified using this method were reviewed for eligibility using the title and abstract and full text review done if they were deemed eligible at this phase. Articles were selected for full text review if they specified invasive bacterial isolates in their abstract. The words used in the search on different sites and unique hits for the word combinations are shown in Table 1 below and flowchart of the process depicted in Fig 1.

**Table 1:** Search strategy.

Database	Search Terms	Studies identified
PubMed	((((((("nigeria"[MeSH Terms] OR "nigeria"[All Fields]) AND ("sepsis"[MeSH Terms] OR "sepsis"[All Fields])) OR ("septicaemia"[All Fields] OR "sepsis"[MeSH Terms] OR "sepsis"[All Fields] OR "septicemia"[All Fields])) OR ("bacteraemia"[All Fields] OR "bacteremia"[MeSH Terms] OR "bacteremia"[All Fields])) AND ("infant, newborn"[MeSH Terms] OR ("infant"[All Fields] AND "newborn"[All Fields]) OR "newborn infant"[All Fields] OR "neonate"[All Fields])) OR ("infant, newborn"[MeSH Terms] OR ("infant"[All Fields] AND "newborn"[All Fields]) OR "newborn infant"[All Fields] OR "neonates"[All Fields])) OR ("infant, newborn"[MeSH Terms] OR ("infant"[All Fields] AND "newborn"[All Fields]) OR "newborn infant"[All Fields] OR "neonatal"[All Fields])) AND ("blood culture"[MeSH Terms] OR ("blood"[All Fields] AND "culture"[All Fields]) OR "blood culture"[All Fields])	4471
AJOL	(Nigeria) AND (Neonate or neonatal or neonates) AND (Sepsis OR septicaemia OR bacteremia)	1123
Google Scholar	(Nigeria) AND (Neonate or neonatal or neonates) AND (Sepsis OR septicaemia OR bacteremia)	8320
	Total	13,914

### Study selection and eligibility criteria

One reviewer reviewed articles and extracted data (NM) based on relevance to the study objectives. A second reviewer crosschecked the articles (KCI) to make sure they were clinically relevant to the review. The two reviewers critically appraised the articles flagged for inclusion and determined by consensus which articles should be included in the systematic review. Both followed guidelines laid out in the PRISMA checklist (S1 Table)[23]. The primary outcome of interest was neonatal sepsis. For evaluation of neonatal sepsis, full length articles were eligible for inclusion if the study was done in Nigeria, evaluated neonates or had extractable neonatal data and specified bacterial isolates obtained from blood cultures. Early onset disease (EOD) and late onset disease (LOD) were defined as sepsis occurring within 1–6 days of life or seven to 28 days of life, respectively, with isolation of the bacterial agent from the blood.

### Quality assessment

The quality of studies was determined based on an appraisal tool developed by Downs and Black [24], and modified to fit the needs of our study as listed in Table 2 below. Because the tool was primarily designed to assess quality of interventional studies, we removed aspects unrelated to interventions because our studies were non-interventional. We thus had nine questions to evaluate out of the 27 proposed by Downs and Black[24]. Our review depended on quality laboratory methods and data, we included four other quality indicators. We thus had 13 indicators to assess quality.

**Table 2:** Criteria selected to determining quality of studies.

Downs and Black modification criteria selected	Other criteria selected by Authors
1. Is the aim of the study clearly defined?	1. Specified participant inclusion and exclusion criteria
2. Are the main outcomes clearly described?	2. Specified a standard method for bacterial identification
3. Are the characteristics of the patients in the study clearly described?	3. Specified eligibility criteria for enrolment
4. Are the main findings of the study clearly described?	4. Reported unadjusted estimates of neonatal sepsis.
5. Were the subjects asked to participate in the study representative of the entire population from which they were recruited?	
6. Were the statistical tests used to analyze the main outcomes appropriate?	
7. Were those subjects who were prepared to participate representative of the entire population from which they were recruited?	
8. Were the staff, places, and facilities where the patients were treated, representative of the treatment the majority of patients receive?	
9. Were the main outcome measures used accurate (valid and reliable)?	

Each item was scored with 1 for ‘yes’ or 0 for ‘no’. Studies which scored 11-13were classified as high quality, those that met eight to ten criteria were scored ‘8–10’ and considered intermediate quality while low quality studies were those meeting less than seven criteria. Low quality studies were eliminated from the review.

### Data extraction

Mendeley^®^ reference manager (London, UK) was used to create a database of studies identified for review. Data such as first author’s name, year of publication, dates and duration of study, study design, sample size, place where study was carried out, dates of study and livebirths during study period, incidence of neonatal sepsis (EOD and LOD), type of blood culture system used, quantity blood collected from neonates, prior antibiotic use before specimen collection for culture and organisms reported were inputed into Excel (Microsoft, Seattle, WA, USA).

### Data analysis

Statistical analysis was performed using Epi-Info version 7.1.4.0 (CDC, Atlanta, GA, USA) and MetaXL version 16.4.3 (EpiGear International Pty Ltd, Wilston, Queensland, Australia). We first assessed the etiology of neonatal sepsis and in a subanalysis the cases caused by GBS. The overall incidence of neonatal sepsis was calculated based on total number of proven cases and number of livebirths at the hospitals of study. The proportion of neonatal sepsis, morbidity, and mortality attributable to GBS and other common neonatal pathogens was determined per 1000 live births. We assessed heterogeneity with Chi-square test and I2 statistic.

## Results

### Study selection and description

In the preliminary search, 13,976 studies were identified from PubMed, AJOL, Google Scholar and bibliography searches out of which 8,862 duplicates were identified, leaving 5,114 manuscripts for initial screening. Of these, 112 were assessed for eligibility based on title and abstract and 24 studies included in the final selection for review (Fig 1). Nine of these also had data that was used to calculate the hospital based incidence of neonatal sepsis in Nigeria.

All 24 selected studies representing 7,802 suspected cases and 2,280 confirmed cases of neonatal sepsis were included. Nine of these studies also had data on hospital based incidence of neonatal sepsis with a total of 31,305 births (Table 3). There was no study reporting community or population-based incidence. Sixteen of the 24 studies, representing 4,699 suspected cases and 1,476 confirmed cases, were conducted in the Southern part of Nigeria while the other eight studies, representing 3,503 suspected cases and 804 confirmed cases, were conducted in Northern Nigeria. Across all studies, a total of 7,802 neonates were suspected of having sepsis and confirmed in 2,280 newborns using blood culture. Selected studies had quality scores ranging from 10–11 (Intermediate to high quality studies). Among studies selected for review of incidence of neonatal sepsis, heterogeneity was high (p<0.0001 and the I2 = 98.2% (95% CI 97.4–98.7).

**Table 3:** Incidence and factors associated with neonatal sepsis.

Study	Quality score	Cases in hospital born	Sample size (Live birth no.)	Location	Healthcare setting	Cultures done	Positive cultures	EOD	LOD	Incidence of Neonatal Sepsis	Culture Method	Previous antibiotic use	Blood volume collected	Mortality rate
Medugu 2017 [25]	11	7	493	Abuja (North)	Tertiary and Secondary	21	7	6	1	14/1000	Automated	No	Weight based criteria	ND
Medugu 2017[26]	11	ND	ND	Abuja (North)	Tertiary	290	81	ND	ND	ND	Automated	ND	1–2 mls	ND
Olatunde 2016[27]	11	ND	ND	Ilesha (South)	Tertiary	306	72	56	16	ND	Manual	No	2–3 mls	25%
Peterside 2015 [28]	11	ND	ND	Bayelsa (South)	Tertiary	223	97	64	33	ND	Manual	No	2–3 mls	8%
Shobowale 2015[29]	11	ND	ND	Lagos (South)	Tertiary	250	85	ND	ND	ND	Automated	ND	1-3mls	15.7
Onyedibe 2015 [30]	11	ND	ND	Jos (North)	Tertiary	218	75	ND	ND	ND	Manual	ND	ND	13.8%
Ekwochi 2014 [31]	10	ND	142	Enugu (South)	Tertiary	44	17	ND	ND	ND	Manual	ND	ND	29.4%
Okon 2014 [32]	11	ND	ND	Maiduguri (North)	Tertiary	1017	70	ND	ND	ND	Manual	ND	2-3mls	ND
Uzodimma 2013 [33]	11	ND	ND	Lagos (South)	Tertiary	39	16	5	11	ND	Automated	Yes (32% across all ages)	2–5 mls	ND
Kingsley 2013 [34]	11	ND	ND	Uyo (South)	Tertiary	357	91	ND	ND	ND	Manual	ND	2-3mls	ND
Awoala + West 2012 [35,36]	11	54	1368	Port Harcourt (South)	Tertiary	406	54	34	20	39.5/1000	Manual	No	2 mls	15%
Ogunlesi 2011[37]	11	174	3390	Sagamu (South)	Tertiary	527	174	119	55	51.3/1000	ND	ND	ND	ND
Nwadioha 2010 [38]	11	ND	ND	Kano (North)	Tertiary	1270	326	ND	ND	ND	Manual	ND	ND	ND
Anah 2008 [6]	10	159	2896	Calabar (South)	Tertiary	717	159	ND	ND	54.9/1000	ND	ND	ND	19.0%
Udo 2008 [39]	10	178	ND	Calabar (South)	Tertiary	215	178	ND	ND	ND	ND	ND	ND	17.1%
Fadero 2007 [40]	11	32	ND	Oshogbo (South)	Tertiary	61	32	10	22	ND	Manual	No	2 mls	31%
Ojukwu 2006 [7]	11	19	2382	Ebonyi (South)	Tertiary	138	33	20	13	7.9/1000	Manual	No	1–2 mls	26.7%
Iregbu 2006 [41]	11	ND	ND	Abuja (North)	Tertiary	390	85	ND	ND	ND	Oxoid Signal	ND	ND	ND
Meremikwu 2005[42]	11	ND	ND	Calabar (South)	Tertiary	533	271	ND	ND	ND	Manual	ND	ND	ND
Mokuolu 2002 [43]	11	29	4118	Ilorin (North)	Tertiary	198	61	30	31	7.0/1000	Manual	No	ND	ND
Adejuyigbe 2001 [44]	10	18	787	Ile-Ife (South)	Tertiary	119	66	35	31	22.9/1000	ND	No	ND	33.3%
Airede 1992 [45]	10	99	15200	Jos (North)	Tertiary	99	99	ND	ND	6.5/1000	Manual	ND	ND	27.3%
Antia-Obong 1991[46]	10	ND	ND	Calabar (South)	Tertiary	275	100	ND	ND	ND	Manual	ND	ND	ND
Owa 1988 [47]	10	9	529	Ilesha (South)	Secondary	89	31	ND	ND	17/1000	Manual	ND	ND	ND
Total		**568**	**31,305**			**7.802**	**2,280**			**18.2/1000**				

Rows in blue indicate data used to derive incidence of neonatal sepsis. ND = Not documented

### Sample sizes and sampling technique

The total number of blood cultures done across the 24 studies was at least 7,802. Ten studies together had performed fewer than 200 blood cultures. The volume of blood collected for culture was reported by 11 studies obtained at least 1 ml of blood from the neonates. One of these studies used new-born weight to determine the volume of blood to collect for culture[25]. Overall blood culture yield across all studies was 24.5% but ranged from 20% [27] to 56% [48]. Eleven studies were retrospective while another 12 were prospective. One study[37] started off using retrospective data and continued with prospectively enrolling participants.

### Source of cases

Twenty-two of the studies were single center studies carried out in tertiary hospitals. Two studies were done in multiple hospitals: one in three and the other in four hospitals[25,49]. Two studies were carried out at different times in the same rural area while all others were in urban areas[27,47]. None took place in a primary health care center.

### Antibiotic use in study population and culture methods

Newborns enrolled in eight prospective studies had specimens taken before commencement of antibiotics. In one prospective study, 33% of newborns had been given at least one antibiotic before enrolment[33]. The BACTEC^®^ blood culture system was used in this study with overall yield of 41%. However, antibiotic use was not documented for 15 studies.

Twenty studies specified the culture technique used for blood culture. Four of them used the BACTEC^®^ continuous monitoring blood culture system [25,26,29,33,50], one used the ‘semi-automated’ Oxoid signal system[41] while others used manual systems using glucose broth, cooked meat broth, tryptone soya broth with sodium polyanathol sulphate or unspecified broths.

### Incidence of neonatal sepsis in general and focus on GBS sepsis

Overall hospital-based incidence of neonatal sepsis reported in the nine selected studies was 18.2/1000 live births (range 7–54.9)[6,7,25,35,37,43,47,48,51] Incidence rates reported ranged from seven to 54.9/1000 live births[6,51] while the incidence of GBS was 0.06/1000 live births. The denominator in all the studies was live births at the hospitals where the studies were carried out. None of the studies reported rates based on live births in the community. To derive the specific incidence of neonatal sepsis arising from GBS in the new-born population, we assessed positive blood cultures yielding GBS among neonates using hospital births as a denominator. Only two studies as shown in Table 4, reported isolation of GBS in neonatal blood cultures (one isolate reported in each study)[7,25]. These were prospective studies and hospital based incidence data was 2/1000 live births for the 2016 study and 0.84/1000 live births for the 2006 study[7,25] as shown in Table 4. One of the studies also evaluated mothers for intrapartum colonization and found the mother of the new-born with invasive disease colonized with the same serotype causing invasive disease in her new-born[25]. Seven studies reported isolation of streptococci but did not further characterize them by serogrouping[6,7,33,35,39,42,46] Two of these specified beta-haemolytic streptococci while the others reported ‘streptococcal species’.

### Etiologies of neonatal sepsis

All 24 studies had bacteriologic profiles comprising a total of 2,280 isolates as detailed in Table 4 below. *S*. *aureus* was isolated in all studies and was the most common isolate in 18 of these, tied with *K*.*pneumoniae* in two of these and was the second most common isolate in the remaining four studies. *K*. *pneumoniae* was isolated in 22 studies and was the most common isolate in four studies where *S*. *aureus* was not the most frequent cause of sepsis[29,30,35,41] *Escherichia coli* was reported in 18 studies and accounted for less than 10% of isolates in 10 of these studies. Six studies reported bacterial agents of neonatal sepsis as either unspecified coliforms or unspecified Enterobacteriaceae. Coagulase negative Staphylococci (CoNS) were reported in 13 studies with four of these reporting isolation rates of more than 10% of the total agents of neonatal sepsis[29,37,43,44]. Notably, 12 studies reported significant isolation of *Pseudomonas spp* (Table 4).

**Table 4:** Aetiological agents of neonatal sepsis in Nigeria.

Study	Positive cultures (n)	GBS (%)	Unspecified Streptococci (%)	*Proteus* spp (%)	*Pseudomonas spp* (%)	*K*. *pneumoniae* (%)	*E*. *coli* (%)	*S*. *aureus* (%)	CoNS* (%)	Others ¶(%)
Medugu 2017 [25]	7	14.3	-	-	-	42.9	-	42.9	-	-
Medugu 2017 [26]	81	-	-	-	8.6	11.1	3.7	59.3	6.2	11.1
Olatunde 2016 [27]	72	-	-	1.4	6.9	11.1	2.8	70	-	7.8
Peterside 2015 [28]	97	_	-	8.2	7.2	14.4	16.5	51.5	-	2.2
Shobowole 2015[29]	85	-	-	4.7	-	36.5	-	18.8	11.1	28.9
Onyedibe 2015 [30]	75	-	-	2.7	4.0	32.0	10.7	30.7	6.7	13.2
Ekwochi 2014 [31]	17	_	-	-	-	-	18	53	-	29
Okon 2014 [32]	70	_	-	1.4	2.9	21.4	8.6	56	1.4	8.3
Uzodimma 2013 [33]	16	_	13	-	-	9	6	56	-	16
Kingsley 2013 [34]	91	-	-	3.3	-	16.5	25.3	42.9	4.4	7.6
Awoala 2012 and West Peterside [35]	169	_	0.9	5.5	3.6	58.2	8.1	20	1.8	1.9
Ogunlesi 2011[37]	174	_	-	6.3	4	23	11	31	12.6	12.1
Nwadioha 2010 [38]	326	_	-	4.4	-	12.3	55.1	27.6	-	0.6
Anah 2008 [6]	363	_	8.3	0.5	2.8	3.3	-	53	-	32.1
Udo 2008 [39]	178	-	8.5	-	-	2.2	-	65.2	-	24.1
Fadero 2007 [40]	32	_	-	18.8	-	9.4	6.3	56.3	6.3	2.9
Ojukwu 2006 [7]	33	3	9.0	3	3	9.1	18.2	45.5	-	12.2
Iregbu 2006 [41]	85	_	-	-	4.7	43.5	1.2	40	2.4	8.2
Meremikwu 2005 [42]	271	_	4.8	0.4	5.9	-	-	51	2.2	35.7
Mokuolu 2002 [43]	61	_	-	-	1.6	16.4	4.9	29.5	24.6	23
Adejuyigbe 2001 [48]	66	_	-	-	18.8	8.7	5.8	36.2	15.9	14.6
Antia-Obong 1992 [52]	100	_	3	1	1	7	3	45	-	40
Airede 1992 [45]	99	-	-	-	-	37		37	-	26
Owa 1988 [47]	31	_	-	-	-	12.9	12.9	25.8	3.2	45.2

* CoNS = Coagulase negative Staphylococci. GBS = Group B Streptococcus

^¶^Other pathogens detected: *Salmonella* spp, *Streptococcus pneumoniae*, *Serratia marcescens*, *Haemophilus influenzae*, *Enterococcus spp*, *Enterobacter spp*.

Twelve studies reported mortality rates attributable to sepsis. The rates varied widely with lowest rate being 8%[28] and highest rate being 33% [44] and an average overall mortality rate of 21.8%.

### Estimates of the burden of GBS-specific sepsis

In our review, the hospital based incidence of neonatal sepsis for all analyzed studies from 1987 to 2017 is 18.3/1000 live births while that of GBS was estimated at 0.06/1000 live births, The most recent census in Nigeria was conducted in,2013 and can only be reliably extrapolated to studies conducted around that time (2011–2015). Hospital based incidence of neonatal sepsis in that period ranged from 39.5–51.3/1000 live births. Seven million annual births occurred in Nigeria from the 2013 census[53] thus, an estimated 276,000–359,100 children will suffer from neonatal sepsis with 420 of these probably attributable to GBS. Based on estimates from other parts of the African continent of a 23% EOD mortality rate and 13% long term sequelae rate respectively[17], an estimated 97 deaths and 55 children with long term disabilities will occur annually in Nigeria from neonatal GBS sepsis.

## Discussion

The incidence of neonatal sepsis at 18.3/1000 live births and that of GBS sepsis at 0.06/1000 live births is significant and warrants active measures to reduce it. These incidence numbers are even likely a gross underestimation of true incidence because of suboptimal health facilities and the relatively small number—36% [53] of deliveries that occur in health care settings in Nigeria. The rapid deterioration in cases of EOD may result in case fatality before arrival and sample collection at the hospital. These cannot be explained merely by differences in isolation techniques or a concentration on hospital case capture as suggested by Dagnew et al[4]. Another postulate is the use of non-prescription antibiotics such as the beta-lactamgroup—which is common in the developing world; these could theoretically target a specific group of bacteria while allowing another group to have a growth advantage. In the long term, this could change the profiles of pathogens colonizing and potentially causing disease in communities.

While guidelines exist in most hospitals about the importance of obtaining blood culture specimen before commencement of antibiotics, in practice, this is often not done for the reasons given in Table 5 below.

**Table 5:** Challenges in isolation of GBS and other pathogens from blood cultures in Nigeria.

	Challenge	Outcome
1.	Lack of continuous availability of blood culture specimen bottles	Missed opportunities for pathogen detection in septic babies is specified in one study [54] but may have also occurred in other studies
2.	Non-availability of critical materials in identification GBS	Reports of isolation of non-specific ‘*streptococci’*, ‘beta haemolytic *streptococci’*, and ‘unidentified bacteria’ with consequent under reporting of GBS isolates.
3.	Requirement of payment for blood culture testing before specimen are obtained.	Inadvertent commencement of antibiotics with resultant reduced yield from blood cultures. Non-collection of specimen with missed opportunities for detection.
4.	Use of manual blood culture systems	Lower sensitivity and lack of antibiotic removal devices result in reduced blood culture yield.

Indeed, it is commonplace to find that the caregivers can’t afford to pay for blood cultures before commencement of antibiotics. The use of manual culture techniques which accounted for 89% of techniques used in this review may have greatly hampered detection of GBS and other pathogens for the following reasons: automated blood culture systems support the growth of a wider range of organisms and at lower inoculum than manual systems, they also have antibiotic removal devices such as resins which help to enhance microbial growth in the presence of antibiotics, the continuous agitation of bottles by the equipment also encourages bacterial growth [55,56]. Dagnew et al reported higher isolation of GBS with automated systems[4].

The large number of cases identified as out-born in the review probably represent a group of babies exposed to antibiotics at home or in other hospitals before referral to study center.

Indeed, in Nigeria a wide range of antibiotics are available without need for prescription. There is no doubt that *E*. *coli*, *K*. *pneumoniae* and *S*. *aureus* overshadow GBS as causes of newborn sepsis in Nigeria, but GBS is the only one at this time with successful prevention strategies in terms of IAP and potential vaccines. There is a need to reduce the incidence of all cause neonatal sepsis and of GBS neonatal sepsis. A potential way to reduce this burden is implementing Intrapartum antibiotic prophylaxis (IAP), this will however be challenging because of the large number of resources and logistics required. In primary and secondary healthcare centres, especially -where the majority of deliveries take place, access to microbiology laboratories which can isolate and identify GBS is minimal, intravenous antibiotics will be logistically difficult to implement and many women traditionally present late in second stage of labour making two doses of IAP impossible to achieve. Although there is a concern that increased use of IAP in obstetric care for GBS prevention will lead to the emergence of antimicrobial resistance among other perinatal pathogens, a study comparing the antibiotic susceptibility of vaginal isolates of *E*. *coli* to ampicillin before and after introduction of IAP found no change[57]. An increase in infections due to other neonatal pathogens has also been postulated but a multi-centre study found no increase overall and an actual decrease in term infants[58].

Vaccination of pregnant women against GBS could be potentially easier as described by Kobayashi et al[59]. The vaccination strategy could also result in reduction in pre-term deliveries and stillbirths. Vaccination against tetanus is well entrenched in the antenatal care protocols across all levelsof health care in Nigeria, thus an additional vaccine would only need to take advantage of the already existing platform which has had a positive outcome worldwide[60], thus the prospect of a GBS vaccine having a similar impact on neonatal health is a realizable outcome. Despite the advantages of a potential GBS vaccine over IAP, it will not be effective at reducing new-born deaths if the prevalent serotypes colonizing and causing invasive disease in this population are not present in the vaccine. There have been several potential GBS vaccines based on the capsular polysaccharide, surface proteins, and polysaccharide–protein conjugates[60]. Antibodies evoked by trial vaccines were able to reach potentially protective levels in new-borns[60,61]. A successful vaccine should prevent more cases of neonatal disease than IAP[62]. While the GBS vaccine may seem like the obvious choice of preventive strategy because it is less logistically challenging[61], timeline of implementation is much longer than IAP–any time in second trimester as against few hours intrapartum, intravenous antibiotics require more skill and consumables than intra venous and could address other problems which IAP does not. It’s potential acceptability will depend on the perceived burden of the disease by health authorities, medical practitioners and the target population especially as other bacterial pathogens are more commonly isolated from neonatal sepsis cases. A recent study found that giving and spreading adequate information is vital in the success of a potential GBS vaccine implementation program[63]. The affordability of such vaccine for economies like Nigeria may be a challenge, and the government is only likely to implement the strategy if its potential impact outweighs the cost. When Kim et al[64] in South Africa evaluated doing nothing, maternal GBS vaccination and IAP, they concluded that compared with doing nothing, the GBS vaccine will cost an average of $1000 per disability-adjusted life-year (DALY) averted while IAP alone would cost $240/DALY. A decision tree model by Russell et al found that a GBS vaccine could prevent a third of GBS related cases and deaths[65].

## Limitations and strengths

Because a number of the reviewed studies were done over a decade ago, the current epidemiologic situation may be somewhat different. Naturally, all the studies reviewed have some bias towards severe disease since all babies reviewed were on admission. We had few indicators of study quality to enable us capture a large number of studies for review. There was substantial heterogeneity across the studies evaluated which makes comparisons difficult. We however did evaluate a wide range of papers using various techniques. To the best of our knowledge, this is the first review of GBS studies in Nigeria, the most populous country in Africa and will serve as a reference source for other reviews and provide guidance for future research.

### Conclusion

Neonatal sepsis is a major cause of morbidity and mortality among newborn in Nigeria. Studies are sparse and concentrated in the Southern part of the country. The average mortality rate of 28% from neonatal sepsis is unacceptable high. Manual methods of culture predominate and identification to specie level lacking for many bacteria especially for streptococci. Although GBS seems to be the focus globally for neonatal sepsis, in Nigeria, the most common pathogens implicated were *S*. *aureus*, *K*. *pneumoniae* and *E*. *coli* with GBS less commonly isolated. There is a dearth of information on the epidemiology of GBS infection in Nigeria. More studies are needed to create a more accurate picture on its burden. We have shown that despite the need for more studies with larger numbers (especially as regards the geographic skewness of data), more robust sampling, sampling techniques and optimal laboratory methods–this burden needs immediate attention. Information about possible hindrances to a potential GBS vaccine will have to be garnered from healthcare providers and pregnant women as a way of potentially encouraging uptake.

## Supporting information

S1 Table PRISMA checklist. (DOC)
